# Unusual paraneoplastic neurological syndrome secondary to a well differentiated pancreatic neuroendocrine tumor: a case report and review of the literature

**DOI:** 10.1186/s12885-015-1923-4

**Published:** 2015-11-18

**Authors:** Maria Pia Brizzi, Cristina Sonetto, Marco Tampellini, Massimo Di Maio, Marco Volante, Giorgio V. Scagliotti

**Affiliations:** 1 Medical Oncology, Azienda Ospedaliera Universitaria San Luigi, Regione Gonzole 10, 10043 Orbassano, Torino Italy; 2 University of Turin, Department of Oncology, Pathology, Azienda Ospedaliera Universitaria San Luigi, Regione Gonzole 10, 10043 Orbassano, Torino Italy; 3 Department of Oncology, University of Turin, Azienda Ospedaliera Universitaria San Luigi, Regione Gonzole 10, 10043 Orbassano, Torino Italy

**Keywords:** Neuroendocrine, Pancreatic tumor, Paraneoplastic neurological syndromes

## Abstract

**Background:**

Paraneoplastic neurological syndrome (PNS) is a heterogeneous group of disorders affecting any part of the nervous system, in a patient affected by cancer. PNS is estimated to occur in 0.01 to 8 % of cancer patients, with higher incidence in those with small cell lung cancer, gynecological tumours or hematological disease. Paraneoplastic cerebellar degeneration (PCD) is the most common PNS, but it has never been reported in patients with pancreatic well-differentiated neuroendocrine tumours.

**Case presentation:**

A 61-year-old man presented with an unusual PNS and absence of circulating neural auto-antibodies. Subsequently, contrast-enhanced computed tomography revealed a large pancreatic mass, together with multiple liver metastases, histologically diagnosed as a well-differentiated neuroendocrine tumor. Initial treatment with long-acting somatostatin analogue (octreotide LAR) and prednisone achieved a biochemical response (reduction of chromogranin A level) and a radiological disease control, but patient experienced only a brief improvement of neurological symptoms. Seven months after the onset of the symptoms, he died from neurological impairment.

**Conclusions:**

PNS can be associated with metastatic non-functioning well-differentiated pancreatic neuroendocrine tumors. These tumors may be unresponsive to treatment with somatostatin analogues and an early neurological treatment should be considered for the optimal management of these uncommon cases.

## Background

Paraneoplastic neurological syndrome (PNS) is a heterogeneous group of disorders affecting any part of the central, peripheral or autonomic nervous system, associated with the presence of a cancer. The etiology of these syndromes has not been fully elucidated yet. Several authors investigated the presence of tumour-associated antibodies against neural antigens (anti-neural antibodies), defining PNS as an immuno-mediated syndrome [1]. However, the absence of anti-neural antibodies does not exclude a diagnosis of PNS, as well as their presence is not sufficient to confirm this diagnosis [2]. This syndrome occurs in 0.01 to 8 % of patients with cancer, and its incidence is higher mainly associated with small cell lung cancer, gynecological tumors as well as hematological diseases [3]. In patients with well-differentiated neuroendocrine tumours PNS has been only occasionally reported.

Paraneoplastic cerebellar degeneration (PCD) is the most common PNS and occurs as a result of autoimmune damage to the cerebellum. It is characterized by subacute cerebellar symptoms and exhibits varying clinical features: In some cases only cerebellar involvement is noted, whereas other sites of the nervous system can be involved in addition to the cerebellum. The syndrome develops within days or a few weeks with dystasia, loss of ambulation, dysarthria, saccadic gaze, pursuit, and nystagmus [3]. Diagnosis is driven by signs and symptoms, because imaging techniques fail to show early abnormalities. Radiological signs of cerebellar atrophy have been reported only months after the clinical onset of the syndrome.

We report the case of a patient with symptoms of sub-acute cerebellar degeneration, in which a pancreatic well-differentiated neuroendocrine tumor was subsequently diagnosed.

## Case presentation

**Fig. 1:**
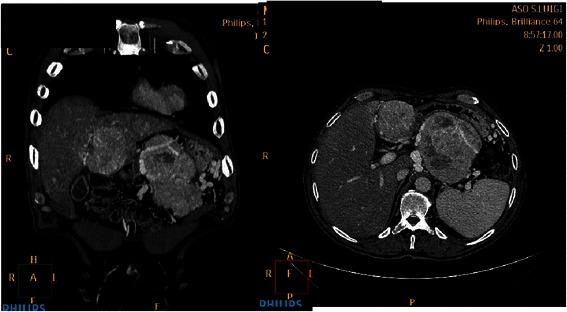
Contrast computed tomography (CT) images of a patient with paraneoplastic cerebellar degeneration due to metastatic pancreatic neuroendocrine tumour

A 61 year-old, Caucasian man, with controlled type II diabetes, came to our attention in April 2011 because of loss of balance that progressed over weeks. There was no family history of neurological or autoimmune disorders. In the preceding month, he started noticing body imbalance, reduced ability to focus on daily activities, to elaborate thoughts, and incoordination. Neurological examination revealed signs associated with acute cerebellar degeneration, such as dysdiadochokinesia, mild dysarthria, dizziness, vertigo and clear ataxia. Baseline International Cooperative Ataxia Rating Scale (ICARS score) [4] was 18. Insulin, gastrin, glucagon, C-peptide, thyroid stimulating hormone, thyroxine, folic acid, vitamin B-12 serum levels and urinary 5-hydroxyindoleacetic acid (5HIAA) levels were normal. Results of the lumbar puncture and lower extremity electromyography were within physiological limits. No brain masses or abnormalities were evident at both magnetic resonance imaging (MRI) and computed tomography (CT) scans. No neural auto-antibodies (anti-Purkinje cells, anti-granule cells, anti-nucleolin, anti-GABAergic synapses, DOT-BLOT IgG - Ravo) were detected, neither in serum nor in cerebro-spinal fluid (CSF). CSF analysis revealed an albumin level of 28,34 mg/dL and an IgG level of 4,50 mg/dL. The cytology of CSF was negative for tumor cells. In May 2011, an abdominal CT scan revealed a large pancreatic mass with multiple liver metastases (Fig. 1). Subsequently, a percutaneous liver biopsy revealed pathological features of well-differentiated neuroendocrine tumor (WDNET) of the pancreas, with a 5 % proliferation index (Ki67). Serum Chromogranin-A was elevated (524 U/l, upper normal limit 18) and the 111In-octreotide scintigram resulted positive. An early treatment with monthly intramuscular octreotide LAR (long-acting releasing) at the dose of 30 mg and daily oral prednisone at the dose of 25 mg were started. A complete disease restaging was performed in September 2011. As expected, the size of the primary lesion and of liver metastases did not change significantly, whereas a good biochemical response was detected, with Chromogranin-A serum level decreasing to 58 U/ml. Clinically, the patient experienced an improvement in neurological symptoms. However, three months later, neurological symptoms rapidly worsened, requiring hospitalization. Electroencephalogram (EEG) showed a typical diffuse encephalopathy pattern, whereas the brain MRI scan was negative. Despite an increase in the dexamethazone daily dose up to 16 mg, during the hospitalization the cerebellar syndrome further deteriorated, preventing patient’s self-care. In October 2011, the ICARS score was 41 and Rankin scale 4 [5]. In November 2011, seven months after the onset of the symptoms, patient died from neurological impairment.

## Discussion

According to diagnostic criteria, the clinical presentation described above is a “classical” PNS. The patient developed a severe pancerebellar syndrome in less than 12 weeks, without evidence of cerebellar atrophy at the MRI, with a progressive deteriorating clinical course, finally dying from neurological impairment. Similarly to what reported in literature [1], in our case the onset of PNS preceded the diagnosis of a cancer. Thus, the presence of clinical findings associated to PNS should focus the diagnostic work-up to search for the presence of a neoplastic disease, even in the absence of any positivity for neural antibodies. Several studies [1, 3] reported cases of PNS in patients concomitantly diagnosed with cancer. The tumors more commonly involved are small cell lung cancer, gynecological cancers, breast cancers, and Hodgkin’s lymphoma [3]. Only few cases of PNS associated with pancreatic WDNET are reported (Table 1) [6–8]. In these cases the treatment of the underling cancer was followed by marked improvement of neurological symptoms.

**Table 1:** Case report of paraneoplastic neurological syndrome associated with well-differentiated pancreatic neuroendocrine tumor

Clinical syndrome	Antibodies	Reference
Lambert-Eaton myasthenic syndrome	Not done	Bertani et al. [6]
Paraneoplastic optic neuropathy	CAR, CRMP-5, anti retinal negative, anti-optic nerve positive	Slamovits et al. [7]
Paraneoplastic encephalomyelitis	Anti Hu, Yo, Ri, Ma1-2, CV2, amphiphysin: negative, GAD positive	Hernández-Echebarría et al. [8]

PCD is the most common PNS representing 24 % of all the PNS, with well-defined criteria [3, 9]. In this case, the onset and the time course of the neurological syndrome fits the diagnosis of PCD, but grossly abnormal EEG and the severity of global encephalopathy leading to death is non typical for this syndrome.

To the best of our knowledge, the association between a pancreatic WDNET and the detection of a PCD has not previously been reported. Neurological impairment and not tumor progression is often the cause of death in these patients. Thus, treatment of PNS is symptomatic and the complete surgical resection of the tumor is highly encouraged [10].

Patients bearing well-differentiated, non-functioning pancreatic neuroendocrine cancers usually present with a slow-growing, less aggressive disease, with life expectancy of more than 24 months [11]. Octreotide LAR is the treatment of choice. In the absence of response, or in case of progression, chemotherapy or everolimus may be considered [12]. These treatments are associated with a disease control (clinical and/or radiological) in more than 50 % of the patients [10]. The case here reported had an atypical - and somehow unexpected - clinical course. After three months of therapy, the patient presented with an improvement of the neurological symptoms and a good biochemical response, with a good correlation between anti-tumor activity and control of clinical symptoms, as usually observed in patients with this type of tumors. Instead, the neurological impairment rapidly progressed independently from the primary tumor response, and precluded the administration of additional treatments. No immunosuppressant agents or treatment with immunoglobulins was administered. This was due to the quick and fatal progression of the neurological symptoms. Furthermore, we recorded the absence of any immunoglobulin in the CSF, so it is questionable whether such therapies would have been beneficial in solving or at least stopping the progressive deterioration of the overall condition.

## Conclusion

This is the first report of an advanced well-differentiated non-functioning neuroendocrine pancreatic tumor with associated an acute paraneoplastic neurological syndrome such as PCD. Neurological symptoms progressed independently from the oncological course of the disease. Thus an early neurological aggressive treatment should be considered, even in the presence of a biochemical or radiological response of the neuroendocrine tumor.

## Consent

Written informed consent for the publication of the clinical details and the radiological images was obtained from the wife of the patient. A copy of the consent form is available for review by the Editor of this journal.

## References

[1] Leypoldt F, Wandinger K-P (2014). Paraneoplastic neurological syndromes. Clin Exp Immunol.

[2] Graus F, Delattre JY, Antoine JC, Dalmau J, Giometto B, Grisold W (2004). Recommended diagnostic criteria for paraneoplastic neurological syndromes. J Neurol Neurosurg Psychiatry.

[3] Dalmau J, Rosenfeld MR (2008). Paraneoplastic syndromes of the CNS. Lancet Neurol.

[4] Trouillas P, Takayanagi T, Hallett M, Currier R, Subramony S, Wessel K (1997). International cooperative ataxia rating scale for pharmacological assessment of the cerebellar syndrome. J Neurol Sci.

[5] Banks JL, Marotta CA (2007). Outcomes validity and reliability of the modified Rankin scale: implications for stroke clinical trials. Stroke.

[6] Bertani H, Messerotti A, Di Benedetto F, Manta R, Greco M, Casoni F (2011). Unusual paraneoplastic syndrome accompanies neuroendocrine tumours of the pancreas. Case Rep Med.

[7] Slamovits TL, Posner JB, Reidy DL, Thirkill CE, Keltner JL (2013). Pancreatic neuroendocrine paraneoplastic optic neuropathy: confirmation with antibody to optic nerve and hepatic metastasis. J Neuroophthalmol.

[8] Hernández-Echebarría L, Saiz A, Arés A, Tejada J, García-Tuñón L, Nieves C (2006). Paraneoplastic encephalomyelitis associated with pancreatic tumor and anti-GAD antibodies. Neurology.

[9] Giometto B, Grisold W, Vitaliani R, Graus F, Honnorat J, Bertolini G (2010). Paraneoplastic neurologic syndrome in the PNS Euronetwork database: a European study from 20 centers. Arch Neurol.

[10] Braik T, Evans AT, Telfer M, McDunn S (2010). Paraneoplastic neurological syndromes: unusual presentations of cancer. A practical review. Am J Med Sci.

[11] Giustina A, Mazziotti G, Maffezzoni F, Amoroso V, Berruti A (2014). Investigational drugs targeting somatostatin receptors for treatment of acromegaly and neuroendocrine tumors. Expert Opin Investig Drugs.

[12] Panzuto F, Rinzivillo M, Fazio N, de Braud F, Luppi G, Zatelli MC (2014). Real-world study of everolimus in advanced progressive neuroendocrine tumors. Oncologist.

